# Associations between cytokine gene polymorphisms and susceptibility to Helicobacter pylori infection and Helicobacter pylori related gastric cancer, peptic ulcer disease: A meta-analysis

**DOI:** 10.1371/journal.pone.0176463

**Published:** 2017-04-28

**Authors:** Jingjing Ma, Dandan Wu, Xue Hu, Jiao Li, Mingwei Cao, Weiguo Dong

**Affiliations:** Department of Gastroenterology, Renmin Hospital of Wuhan University, Wuhan, Hubei Province, China; Oita University Faculty of Medicine, JAPAN

## Abstract

**Objectives:**

The aim of this study is to clarify the associations between IL-1B31C/T, IL-1B-511C/T, IL-8-251T/A gene polymorphisms and the risk of Helicobacter pylori (*H*. *pylori*) infection together with *H*. *pylori*-related gastric cancer (GC), peptic ulcer disease (PUD).

**Methods:**

All eligible literature published up to July 2016 were identified by searching Pubmed, Embase, Web of Science and CNKI. Pooled odds ratio (OR) and 95% confidence interval (95% CI) were calculated using a fixed or random effects model.

**Results:**

29 case-control studies were eligible, and each of them may focus on more than one gene polymorphism. Ultimately, there were 21 studies (3159 cases and 2816 controls) for IL-1B-31C/T, 16 studies (2486 cases and 1989 controls) for IL-1B-511C/T polymorphisms, 9 studies (1963 cases and 1205 controls) for IL-8-251T/A polymorphisms. Overall, an increased risk of *H*. *pylori* infection was found for IL-1B-31C/T polymorphisms in total population [OR = 1.134, 95%CI = 1.008–1.275 for recessive model; OR = 1.145, 95%CI = 1.007–1.301 for TT *vs* CC model]. While, for IL-1B-511C/T and IL8-251T/A polymorphisms, no evidence indicated that they were associated with the risk of *H*. *pylori* infection in all genetic models. Furthermore, we found an increased risk of *H*. *pylori*-related GC with IL-1B-511C/T polymorphisms [OR = 1.784, 95%CI = 1.289–2.469 for recessive model; OR = 1.772, 95%CI = 1.210–2.594 for TT *vs* CC model] and IL8-251A/T polymorphisms [OR = 1.810, 95%CI = 1.229–2.667 for recessive model; OR = 1.717, 95%CI = 1.143–2.580 for TT *vs* AA model], an increased risk of H. pylori-related PUD with IL8-251T/A polymorphisms [OR = 1.364, 95%CI = 1.010–1.843 for recessive model; OR = 1.427, 95%CI = 1.039–1.959 for AA *vs* TT model].

**Conclusions:**

IL-1B-31C/T gene polymorphisms might increase *H*. *pylori* infection risk. IL-1B-511-C/T and IL-8-251T/A gene polymorphisms might act as a risk factor to H. pylori-related diseases including GC or PUD

## Introduction

Helicobacter pylori (*H*. *pylori*) is well known as a special bacterium that usually establishes in the human stomach, which is a special etiological factor of various gastro-duodenal diseases, including chronic gastritis, peptic ulcer disease (PUD) including gastric ulcer and duodenal ulcer, some forms of gastric cancer(GC), even colonic cancer and pancreatic cancer[1,2,3,4]. However, in most cases the *H*. *pylori* infection is asymptomatic [5]. Pathogenic factors of *H*. *pylori* infection and the development of H. pylori-related gastric diseases were influenced by the degree of virulence of the *H*. *pylori* strain, the host genetic susceptibility and environmental factors [6,7]. Increasing researches indicated that genetic factors that regulate cytokine production can effect an individual's susceptibility to *H*. *pylori* infection, which plays a major role in the pathogenic process of *H*. *pylor*i-related diseases such as PUD, GC [8,9]. The expression of pro-inflammatory cytokine creates a condition of hypoacidity that favors the survival and colonization of *H*. *pylori* [10,11].

IL-1β (encoded by IL-1B gene) is involved in many cellular activities including inflammatory response and secretion of gastric acid [12,13]. It has been shown IL-1B-31C/T and IL-1B-511C/T polymorphisms are closely related to GC, they are found to more frequently occur in Chinese GC patients [14,15]. *H*. *pylori* infection may have an interactive relationship with IL-1β gene. *H*. *pylori* infection induces IL-1B expression, increasing the mucosal IL-1β levels, while the IL-1β levels decreases after *H*. *pylori* eradication[16,17]. In addition, the -31C allele of IL-1B seems to be as a potent depressor of gastric acid secretion, IL-1β is a 100-fold more potent inhibitor than PPIs, and 6000-fold more potent than H2 receptor antagonists on a molar basis, which allows the expansion and reproduction of *H*. *pylori* colonization[18].

It has been reported that IL-8 gene has a polymorphism of an A/T base pair in the promoting region (−251), which is strongly associated with increasing the synthesis of interleukin by gastric epithelial cells [19,20,21]. IL-8 is a pro-inflammatory chemokine and plays an important role in the pathogenesis of H. pylori-induced diseases[22]. A powerful and strong evidence has shown that, during *H*. *pylori* infection, particularly the cag-PAI-positive strain of *H*. *pylori*, high expression of IL-8 from gastric epithelial cells plays an important role in the initiation, modulation and maintenance of inflammatory responses, which can amplify the inflammatory responses via recruiting neutrophils and monocytes, leading to a development degree of gastritis [23]. It is also reported that IL8-251TT genotype seems to act as a protective factor against *H*. *pylori* infection while IL8-251TA genotype may increase the risk of *H*. *pylori* infection [24]. Ivy Bastos Ramis reported that *H*. *pylori* infection patients with the A/A genotype at the IL-8-251 position have an increased risk of PUD [25].

Although many previous studies have concentrated the association of H. pylori infection or H. pylori-related diseases with the IL-1B-31C/T, IL-1B-511C/T, IL-8-251T/A polymorphism, they drew complicated and inconsistent results. Zhao Y et al [26] reported that no statistical significance was found about the correlation between IL-1B-31C/T and *H*. *pylori* infection, but one recent meta-analysis suggested that IL-1B-31C/T polymorphism might confer susceptibility to *H*. *pylori*-related GC [27]. Caleman Neto A et al [24] revealed that it was IL-8-251T/A polymorphism associated with *H*. *pylori* infection instead of IL-1B-511 C/T polymorphisms, but Park MJ [28] suggested that *H*. *pylori* infection might increase the association between IL-1B-511C/T and stomach carcinoma. Additionally, an interesting observation has shown that the IL1B-511TT genotype was associated with the risk of PUD and *H*. *pylori* [29]. The results remain inconclusive and a comprehensive analysis is necessary. Therefore, we conducted this meta-analysis to clarify the associations between IL-1B31C/T, IL-1B-511C/T, IL-8-251T/A gene polymorphisms and the risk of *H*. *pylori* infection together with *H*. *pylori*-related GC and PUD.

## Materials and methods

### Search strategy

In this work, we conducted a meta-analysis about the association between IL-1, IL-8 gene polymorphisms and H. pylori infection. This study was approved by Wuhan University Renmin Hosipital. A systematic literature search was conducted for articles about IL-1B31C/T, IL-1B-511C/T, IL-8-251T/A gene polymorphism associated with *H*. *pylori* infection or *H*. *pylori*-related disease in a manner with combination of free terms and subject terms. Relevant researches were searched using the terms [interleukin or cytokine or IL] AND [gene] AND [variants or polymorphism or single nuclear polymorphism or mutation] AND [Helicobacter pylori infection or Helicobacter pylori-related diseases or *H*. *pylori* or *H*. *pylori*-related diseases] via the Pubmed, Embase, Chinese National Knowledge Infrastructure (CNKI), Web of Science Databases without the language restriction. The search was restricted to humans. Additional studies were acquired by screening references in the retrieved articles and preceding reviews on the topic.

### Study selection

The studies included must meet the following criteria: 1) case-control studies, 2) described the association of IL-1B31C/T, IL-1B-511C/T, IL-8-251T/A gene polymorphism with *H*. *pylori* infection, 3) studies had detailed the numbers of both controls and cases so that we could obtain available genotype frequencies. Accordingly, the exclusion criteria: 1) case-only studies, case reports, and review articles, 2) duplicate data, 3) only for benign disease compared with controls, 4) studies that investigated target gene variants as marks for response to therapy.

### Data extraction and quality assessment

Two investigators selected the article and extracted the data independently, reaching a consensus on all of the terms. If they generated different results, it’s necessary to check the data again and have a discussion until an agreement. If they could not reach an agreement, an expert (Dong WG) would join in the discussion to reach an agreement. The data extracted from any article included the first author’s name, the publication year, country, ethnicity, study design, genotype method, the number of case and control. The ethnicities were classified as Asian or Caucasian population. The quality of selected studies was independently evaluated on basis of Newcastle-Ottawa scale (NOS) [30]. Studies with six or more stars were considered as high quality.

### Statistical analysis

The meta-analysis was performed using the Cochrane Collaboration Revman 5.2 and STATA package version 11.0 (Stata corporation, College Station, Texas). The risk of *H*. *pylori* infection associated with IL-1B31C/T, IL-1B-511C/T, IL-8-251T/A gene polymorphism was estimated for each eligible study by odd ratios (OR) corresponding to a 95% confidence interval (95% CI). A χ^2^ -based Q statistical test and *I*^2^ index were performed to assess the between-study heterogeneity [31,32]. If P < 0.10 or I^2^ > 50%, the ORs were pooled using a random effect model. Hardy-Weinberg equilibrium (HWE) in control people was judged by χ^2^ test. We evaluated the associations of IL-1B31C/T, IL-1B-511C/T, IL-8-251T/A gene polymorphisms with *H*. *pylori* infection under dominant, recessive, co-dominant, and heterozygote models. Then, stratification analyses were further performed on ethnicity, study design, GC and PUD. Analysis of sensitivity was performed to evaluate the stability of the results. Finally, Publication bias was diagnosed with Begg’s funnel plot and Egger’s linear regression [33,34]. P < 0.05 was considered as statistically significant.

## Results

### Study characteristics

**Fig 1:**
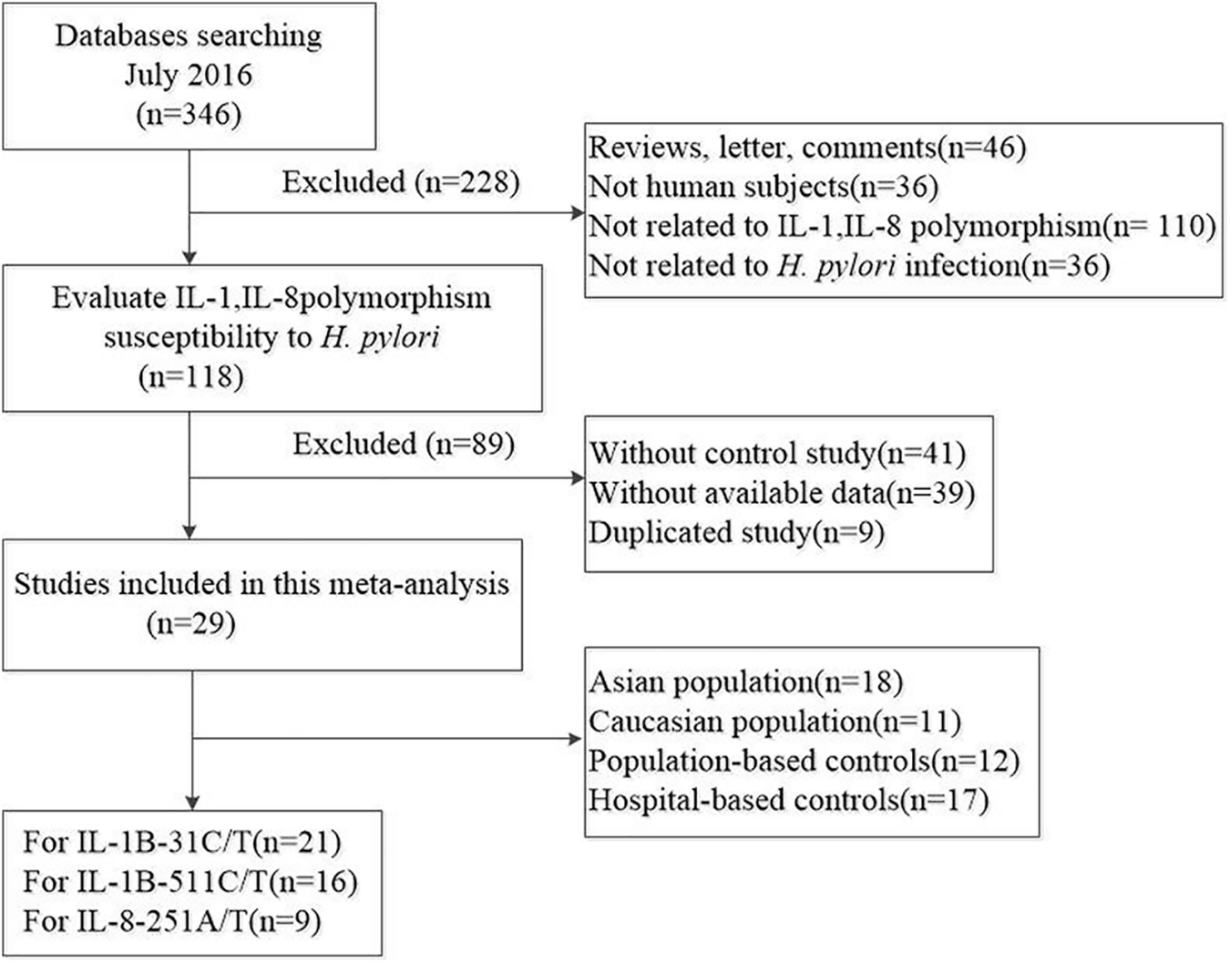
The flow chart of study selection.

346 potentially relevant studies were retrieved by our search strategy. According to the inclusion and exclusion criteria, 29 studies with full text were ultimately eligible for this meta-analysis and 317 studies were excluded. The flow chart of study selection is summarized in Fig 1. Each of eligible studies may focus on more than one gene polymorphism, and even more than one group in the research. Ultimately, there were twenty-one studies with 3159 cases and 2816 controls concerning IL-1B-31C/T gene polymorphisms, sixteen studies with 2486 cases and 1989 controls concerning IL-1B-511C/T gene polymorphisms, nine studies with 1963 cases and 1205 controls concerning IL-8-251T/A gene polymorphisms. The major characteristics of the included studies are summarized in Table 1. Ethnicity include Asian and Caucasian, two type of study design mean hospital-based population (HB) and Publication-based (PB). PCR-RFLP, PCR-CTPP or Taqman were performed in including studies. Among the enrolled studies, three studies focus on the risk of *H*. *pylori* infection GC patients for IL-1B-31C/T, six studies focus on the risk of GC in *H*. *pylori* infection individuals for IL-1B-511C/T, three focus on the risk of GC in *H*. *pylori* infection individuals for IL-8-251T/A, five focus on the risk of PUD in *H*. *pylori* infection individuals for IL-8-251T/A. Blood samples were used to determine genetic polymorphisms in all of the included studies. The distribution of genotype in the controls was consistent with HWE for the selected studies except only one study (P_HWE_ was shown in the Table 1). The qualities of all enrolled studies were categorized as high quality according to the NOS score shown in the Table 1.

**Table 1:** Charateristics of studies included in the meta-analysis.

Study (author)	Year	Country	Ethnicity	Design	Genotype Method	Number	P_HWE_	NOS score
						Case/Control		
IL-1B-31C/T								
Abdiev S	2010	Uzbeks	Caucasian	PB	PCR-CTPP	124/42	0.534	6
Caleman Neto A	2014	Brazil	Caucasian	PB	PCR-RFLP	30/30	0.876	6
Hamajima N	2002	Japan	Asian	PB	PCR-CTPP	324/207	0.070	7
Hamajima N(1)	2001	Japan	Asian	HB	PCR-CTPP/RFLP	82/36	0.863	6
Hamajima N(2)	2001	Japan	Asian	HB	PCR-CTPP/RFLP	69/54	0.669	6
He BS (1)	2011	China	Asian	HB	PCR-RFLP	251/141	0.505	6
He BS (2)	2011	China	Asian	HB	PCR-RFLP	240/268	0.299	6
Hu S	2005	China	Asian	PB	PCR-RFLP	96/159	0.650	7
Kang WK	2004	Korea	Asian	HB	PCR-RFLP	33/68	0.977	6
Katsuda	2003	Japan	Asian	PB	PCR-CTPP	242/195	0.431	7
Kumar S(1)	2009	India	Caucasian	HB	PCR-RFLP	111/25	0.922	7
Kumar S(2)	2009	India	Caucasian	HB	PCR-RFLP	56/54	0.058	7
Li J	2010	China	Asian	HB	PCR-RFLP	53/75	0.554	6
Queiroz DM	2009	Brazil	Caucasian	HB	PCR-CTPP	370/171	0.222	7
Saijo Y	2007	Japan	Asian	PB	TaqMan	237/173	0.666	7
Tseng FC	2006	Jamaica	Asian	HB	TaqMan	36/147	0.928	8
Uno M (1)	2002	Brizal	Caucasian	PB	PCR-CTPP	196/198	0.163	7
Uno M (2)	2002	Brizal	Caucasian	PB	PCR-CTPP	261/292	0.061	7
Yang J(1)	2004	China	Asian	PB	PCR-RFLP	151/126	0.722	8
Yang J(2)	2004	China	Asian	PB	PCR-RFLP	164/94	0.414	8
Zhao Y	2013	Indonesia	Asian	PB	PCR-CTPP	33/261	0.889	7
IL-1B-511C/T								
Arango MT	2010	Colombia	Caucasian	HB	PCR-CTPP/RFLP	66/45	0.295	7
Caleman Neto A	2014	Brazil	Caucasian	PB	PCR-RFLP	30/30	0.543	6
Hamajima N	2001	Japan	Asian	HB	PCR-CTPP/RFLP	149/90	0.414	6
He BS	2011	China	Asian	HB	PCR-RFLP	491/409	0.52	6
Hu S	2005	China	Asian	PB	PCR-RFLP	96/159	0.858	6
Jiang	2007	China	Asian	HB	PCR-RFLP	118/50	0.586	7
Kang WK	2004	Korea	Asian	HB	PCR-RFLP	33/68	0.977	6
Kimang'a AN	2012	Kenya	Caucasian	HB	PCR-RFLP	151/119	0.096	7
Kumar S	2009	India	Caucasian	HB	PCR-RFLP	167/79	0.195	6
Li C	2007	China	Asian	HB	PCR-RFLP	374/289	0.973	7
Li Q	2010	China	Asian	HB	PCR-RFLP	182/44	0.25	7
Saijo Y	2007	Japan	Asian	PB	TaqMan	237/173	0.518	7
Tseng FC	2006	Jamaica	Asian	HB	TaqMan	34/149	0.283	7
Yakar T	2015	Turkey	Caucasian	HB	PCR-RFLP	66/45	0.295	7
Zeng ZR (1)	2003	China	Asian	HB	PCR-RFLP	135/142	0.46	8
Zeng ZR (2)	2003	China	Asian	HB	PCR-RFLP	157/98	0.069	8
IL-8-251T/A								
Abdiev S	2010	Uzbeks	Caucasian	PB	PCR-CTPP	124/42	0.031	6
Caleman Neto A	2014	Brazil	Caucasian	PB	PCR-RFLP	30/30	0.218	6
Chakravorty M	2008	India	Caucasian	HB	PCR	153/157	0.998	6
Farshad S	2010	Iran	Asian	HB	PCR-RFLP	90/371	0.587	7
Kang JM	2009	Korea	Asian	PB	PCR	884/160	0.478	7
Kumar S	2015	India	Caucasian	PB	PCR-ARMS	394/82	0.898	8
Qadri Q	2014	Kashmir	Asian	PB	PCR-CTPP	104/26	0.993	7
Ramis IB	2015	Brazil	Caucasian	HB	PCR-RFLP	151/76	0.754	7
Zhao Y	2013	Indonesia	Asian	PB	PCR-CTPP	33/261	0.127	7

HWE, Hardy–Weinberg equilibrium, P_HWE_, was calculated by goodness-of fit chi-square test, P_HWE_<0.05 means the controls of studies wasn’t in agreement with HWE.

PB, population-based controls.

HB, hospital-based controls.

### Quantitative data synthesis

**Fig 2:**
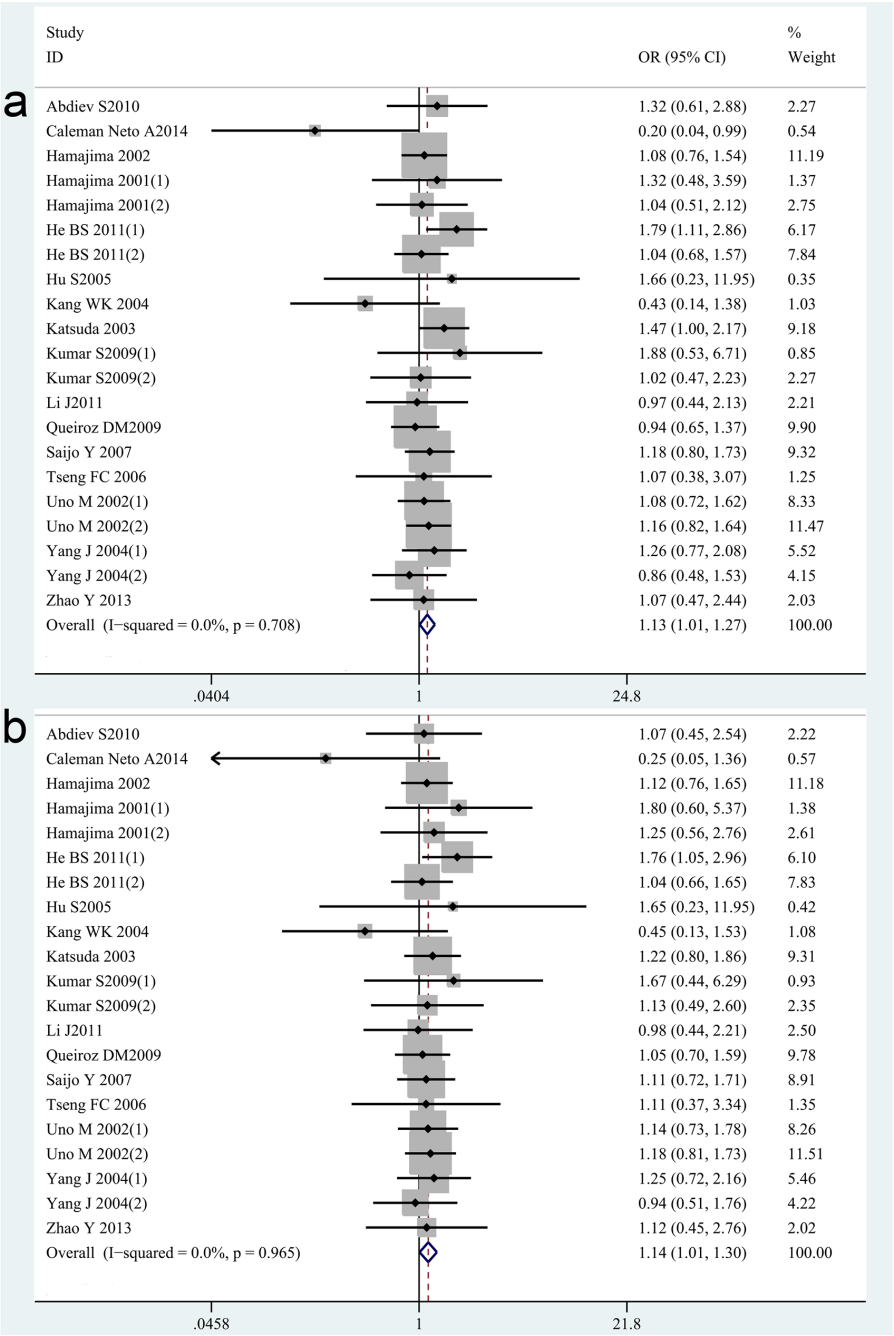
Meta-analysis of the association between IL-1B-31C/T polymorphisms and susceptibility to *H*. *pylori* infection. (a). Recessive model. (b) TT vs CC model.

For IL-1B-31C/T, twenty-one case-control studies with 3159 cases and 2816 controls concerning IL-1B-31C/T gene polymorphisms were identified [15,24,26,35–47]. Overall, there is a significant difference in IL-1B-31C/T genotype distribution between *H*. *pylori* infection and control [OR = 1.13, 95%CI = 1.01–1.28 for recessive model, p = 0.04; OR = 1.15, 95%CI = 1.01–1.30 for TT vs CC model, p = 0.04] (Fig 2). Furthermore, a significant association was also found in subgroup analysis under recessive model based on Asian population [OR = 1.18, 95%CI = 1.02–1.37, p = 0.03] and GC group [OR = 1.54, 95%CI = 1.11–2.13, p = 0.01], but not in the Caucasian population [OR = 1.06, 95%CI = 0.87–1.29, p = 0.56] (Table 2). Meantime, the association between IL-1B-31C/T gene polymorphisms and *H*. *pylori* infection seems not to be related to the subgroup analysis based on study design including PB and HB under all genetic compared model (shown in the Table 2).

**Table 2:** Summary of ORs of the IL-1B-31C/T, IL-1B-511C/T, IL-8-251A/T gene polymorphisms and H. pylori infection.

Gene	N	Dominant model				Recessive model				Homozygous model				Heterozygous model			
		OR(95%CI)	P^a^	P^b^	I^2^	OR(95%CI)	P^a^	P^b^	I^2^	OR(95%CI)	P^a^	P^b^	I^2^	OR(95%CI)	P^a^	P^b^	I^2^
IL-1B-31C/T																	
Total	21	1.06(0.97,1.15)	0.20	0.29	13%	1.13(1.01,1.28)	0.04	0.15	25%	1.15(1.01,1.30)	0.04	0.19	32%	1.06(0.96,1.18)	0.24	0.99	0%
Asian	14	1.06(0.96,1.17)	0.28	0.14	30%	1.18(1.02,1.37)	0.03	0.22	21%	1.17(0.99,1.37)	0.06	0.92	0%	1.06(0.94,1.20)	0.36	0.35	9%
Caucasian	7	1.05(0.91,1.21)	0.49	0.62	0%	1.06(0.87,1.29)	0.56	0.18	32%	1.11(0.89,1.37)	0.37	0.22	28%	1.07(0.89,1.27)	0.47	0.75	0%
PB	11	1.04(0.94,1.16)	0.422	0.86	0%	1.15(0.99,1.34)	0.06	0.24	21%	1.13(0.96,1.32)	0.14	0.45	0%	1.04(0.91,1.19)	0.55	0.88	0%
HB	10	1.07(0.94,1.16)	0.30	0.05	47%	1.10(0.91,1.34)	0.33	0.14	34%	1.17(0.95,1.45)	0.14	0.71	0%	1.10(0.93,1.29)	0.26	0.26	20%
GC ^c^	3	1.14(0.91,1.43)	0.24	0.88	0%	1.54(1.11,2.14)	0.01	0.43	0%	1.51(1.05,2.17)	0.03	0.66	0%	1.14(0.87,1.49)	0.34	0.83	0%
IL-1B-511C/T																	
Total	16	1.02(0.93,1.12)	0.71	0.33	11%	1.03(0.90,1.19)	0.66	0.54	0%	1.04(0.89,1.21)	0.64	0.54	0%	1.02(0.91,1.14)	0.70	0.22	21%
Asian	11	1.03(0.92,1.14)	0.65	0.41	3%	0.99(0.85,1.16)	0.92	0.32	13%	1.03(0.86,1.22)	0.77	0.35	9%	1.04(0.92, 1.17)	0.55	0.45	0%
Caucasian	5	0.99(0.80,1.23)	0.92	0.19	35%	1.19(0.88,1.61)	0.26	0.99	0%	1.08(0.76,1.50)	0.65	0.67	0%	0.95(0.73,1.24)	0.71	0.11	46%
IL-8-251T/A																	
Total	9	1.01(0.88,1.17)	0.86	0.15	34%	1.12(0.91,1.39)	0.28	0.15	34%	1.10(0.88,1.37)	0.43	0.11	39%	0.99(0.83,1.17)	0.87	0.17	31%
Asian	4	0.95(0.78,1.16)	0.64	0.28	21%	1.10(0.69,1.76)	0.69	0.07	58%	1.00(0.63,1.60)	0.99	0.04	64%	0.91(0.72,1.14))	0.40	0.64	0%
Caucasian	5	1.07(0.88,1.31)	0.48	0.32	15%	1.11(0.84,1.46)	0.46	0.29	19%	1.13(0.85,1.52)	0.40	0.41	0%	1.09(0.85,1.40)	0.50	0.22	31%

N, number of studies.

OR, odds ratio; 95%CI, 95% confidence interval.

^a^ Text for overall effect.

^b^ Text for heterogeneity, a random effects model was used when the P for heterogeneity test < 0.10 or I2 > 50%, otherwise, a fixed effects model was used.

^c^ In this subgroup, the H. pylori infection individuals were from gastric cancer patients.

**Fig 3:**
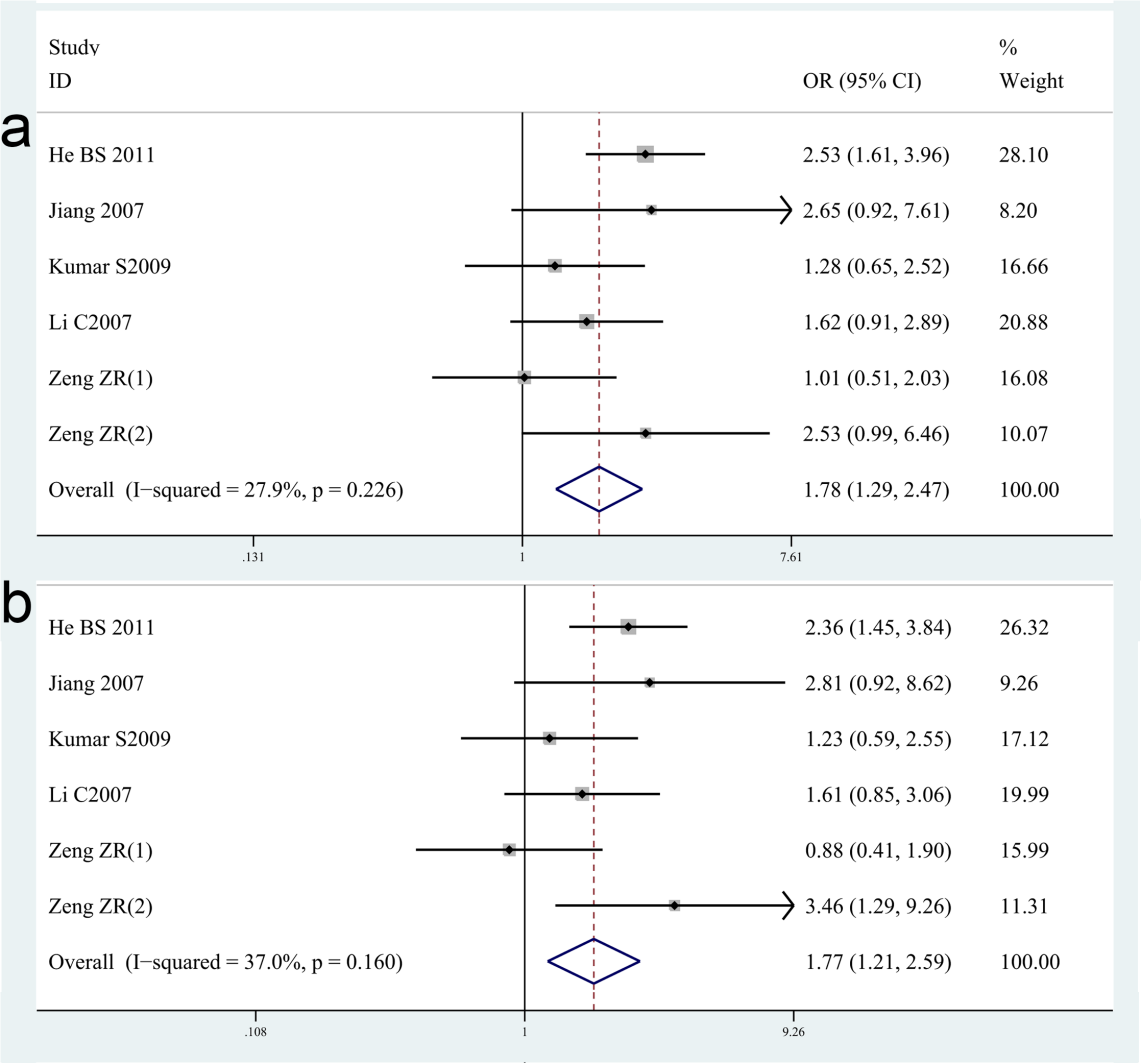
Meta-analysis of the association between IL-1B-511C/T polymorphisms and susceptibility to *H*. *pylori*-related GC. (a). Recessive model. (b) TT vs CC model.

For IL-1B-511C/T, sixteen studies with 2486 cases and 1989 controls concerning IL-1B-511C/T gene polymorphisms were identified [15,24,37–39,41,44,45,48–54].Overall, there is no significant difference in IL-1B-511C/T genotype distribution between *H*. *pylori* infection and control, neither in various subgroup analysis including ethnicity and study design [OR = 1.02, 95%CI = 0.93–1.12 for dominant model, p = 0.71; OR = 1.03, 95%CI = 0.90–1.19 for recessive model, p = 0.66] (Table 2). While, six studies with 647 *H*. *pylori*-related GC cases and 660 *H*. *pylori* inflection controls concerning IL-1B-511-C/T gene polymorphisms were further identified [15,41,49,51,54]. We found an increased risk of *H*. *pylori*-related GC with IL-1B-511C/T polymorphisms [OR = 1.78, 95%CI = 1.29–2.47 for recessive model, p<0.001; OR = 1.77, 95%CI = 1.21–2.59 for TT vs CC model, p = 0.003] (Fig 3, Table 3).

**Table 3:** Summary of ORs of IL-1B-511C/T, IL-8-251T/A gene polymorphisms and H. pylori-related GC and PUD risk.

Gene	N	Dominant model				Recessive model				Homozygous model				Heterozygous model			
		OR(95%CI)	P^a^	P^b^	I^2^	OR(95%CI)	P^a^	P^b^	I^2^	OR(95%CI)	P^a^	P^b^	I^2^	OR(95%CI)	P^a^	P^b^	I^2^
IL-1B-511C/T																	
GC	6	1.21(1.02,1.43)	0.03	0.68	13%	1.78(1.29,2.47)	0.00	0.23	28%	1.77(1.21,2.59)	0.00	0.16	37%	1.20(0.98,1.47)	0.08	0.71	0%
IL-8-251T/A																	
GC	3	1.13(0.90,1.41)	0.29	O.88	0%	1.81(1.23,2.67)	0.00	0.88	0%	1.72(1.14,2.58)	0.01	0.86	0%	1.08(0.84,1.39)	0.54	0.731	0%
PUD	5	1.15(0.96,1.37)	0.13	0.99	0%	1.36(1.01,1.84)	0.04	0.53	0%	1.43(1.04,1.96)	0.03	0.60	0%	1.15(0.94,1.41)	0.18	0.996	0%

N, number of studies.

OR, odds ratio; 95%CI, 95% confidence interval.

^a^ Text for overall effect.

^b^ Text for heterogeneity, a random effects model was used when the P for heterogeneity test < 0.10 or I2 > 50%, otherwise, a fixed effects model was used.

**Fig 4:**
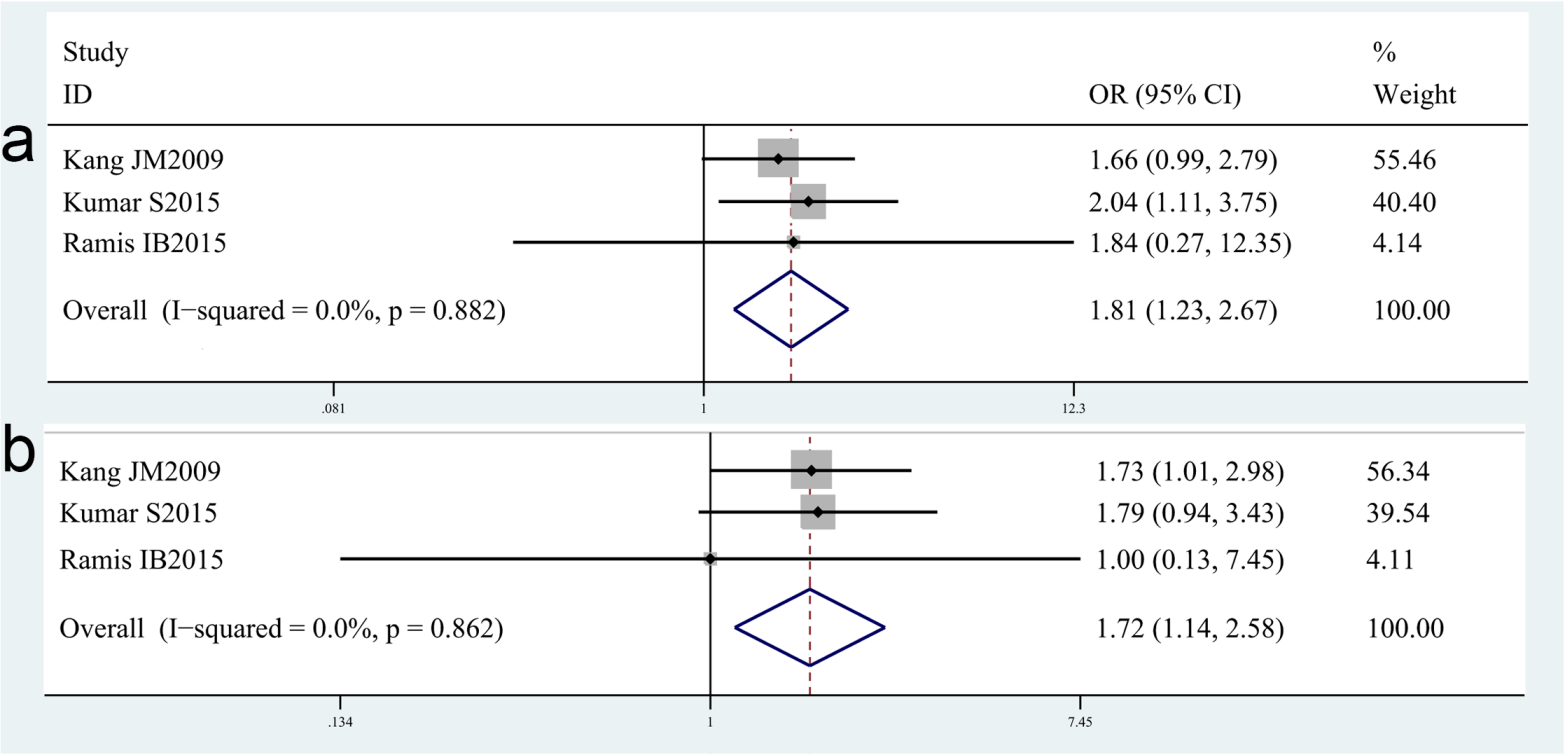
Meta-analysis of the association between IL-8-251T/A polymorphisms and susceptibility to *H*. *pylori*-related GC. (a). Recessive model. (b) TT vs AA model.

**Fig 5:**
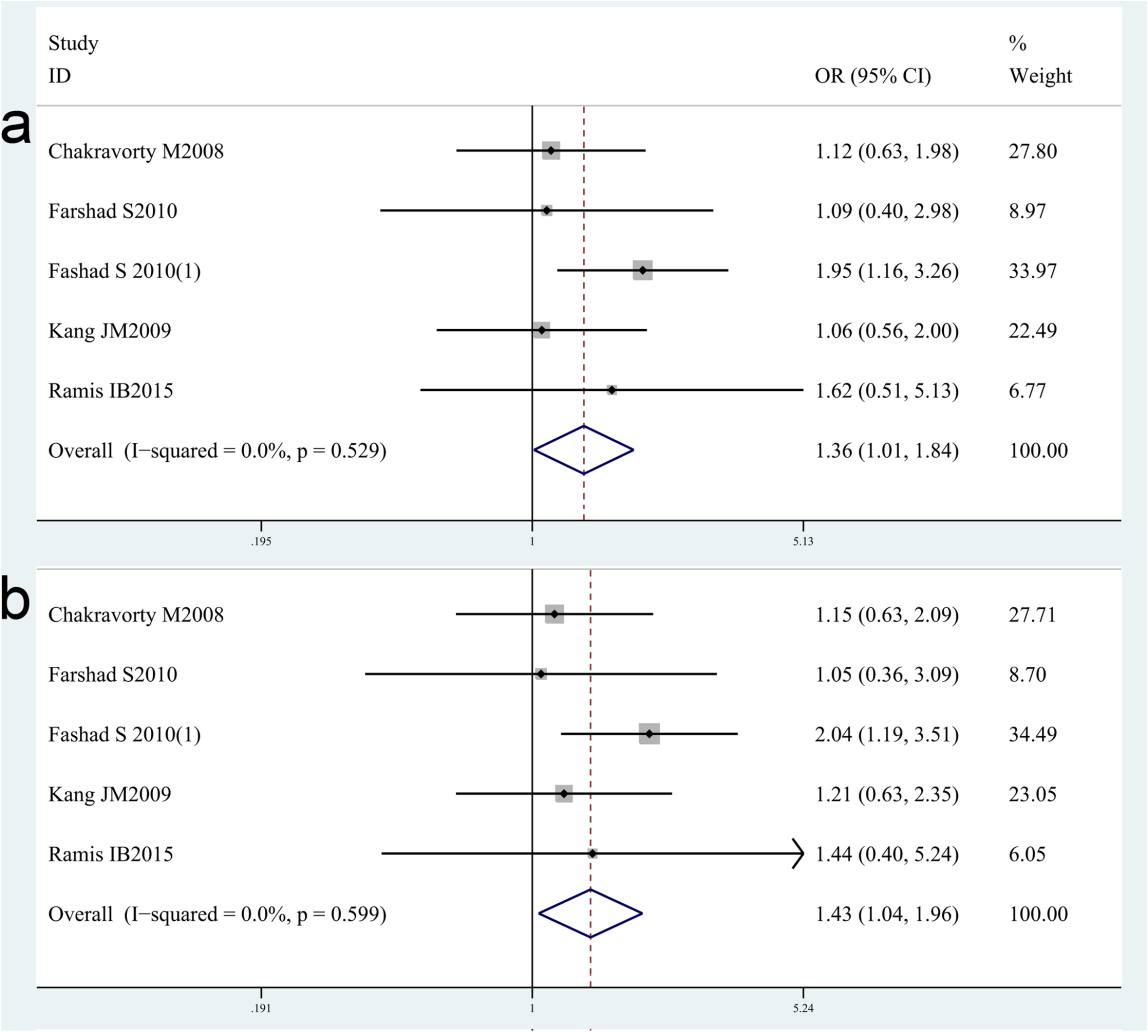
Meta-analysis of the association between IL-8-251T/A polymorphisms and susceptibility to *H*. *pylori*-related PUD. (a). Recessive model.(b) TT vs AA model.

For IL-8-251T/A, nine studies with 1963 cases and 1205 controls concerning IL-8-251T/A gene polymorphisms were identified [24,25,35,55–60]. Overall, there is no significant difference in IL-8-251C/T genotype distribution between *H*. *pylori* infection case and control[OR = 1.01, 95%CI = 0.88–1.17 for dominant model, p = 0.86; OR = 1.12, 95%CI = 0.91–1.39 for recessive model, p = 0.28], neither in various ethnicity-based subgroup analysis including Asian population [OR = 0.95, 95%CI = 0.78–1.16 for dominant model, p = 0.64; OR = 1.10, 95%CI = 0.69–1.76 for recessive model, p = 0.69]and Caucasian population[OR = 1.07, 95%CI = 0.88–1.31 for dominant model, p = 0.48; OR = 1.11, 95%CI = 0.84–1.46 for recessive model, p = 0.46] (shown in the Table 2). While, three studies [25,58,59]focus on the risk of *H*. *pylori*-related GC and five studies [25,56,57,58] focus on the risk of *H*. *pylori-*related PUD for IL-8-251T/A gene polymorphisms. The results are rather interesting and exciting. We found an increased risk of *H*. *pylori*-related GC with IL-8-251T/A polymorphisms [OR = 1.81, 95%CI = 1.23–2.67 for recessive model, p = 0.003; OR = 1.72, 95%CI = 1.14–2.58 for TT vs AA model, p = 0.01](Fig 4,Table 3), an increased risk of *H*. *pylori*-related PUD with IL-8-251T/A polymorphisms[OR = 1.36, 95%CI = 1.01–1.84 for recessive model, p = 0.04; OR = 1.43, 95%CI = 1.04–1.96 for AA vs TT model, p = 0.03] (Fig 5,Table 3).

### Heterogeneity and sensitivity analysis

The heterogeneity of each included studies pertaining to each polymorphism is clearly presented in the Table 2 and Table 3. No significant heterogeneity was observed between enrolled studies in all comparisons under each model (P>0.05, or I^2^<50%).Then, sensitivity analysis was performed to evaluate the stability of the results by removing one study one by one. The estimated pooled odd ratio did not change after removing any single study. The above analysis indicated that the results were stable and statistically robust.

### Publication bias

**Fig 6:**
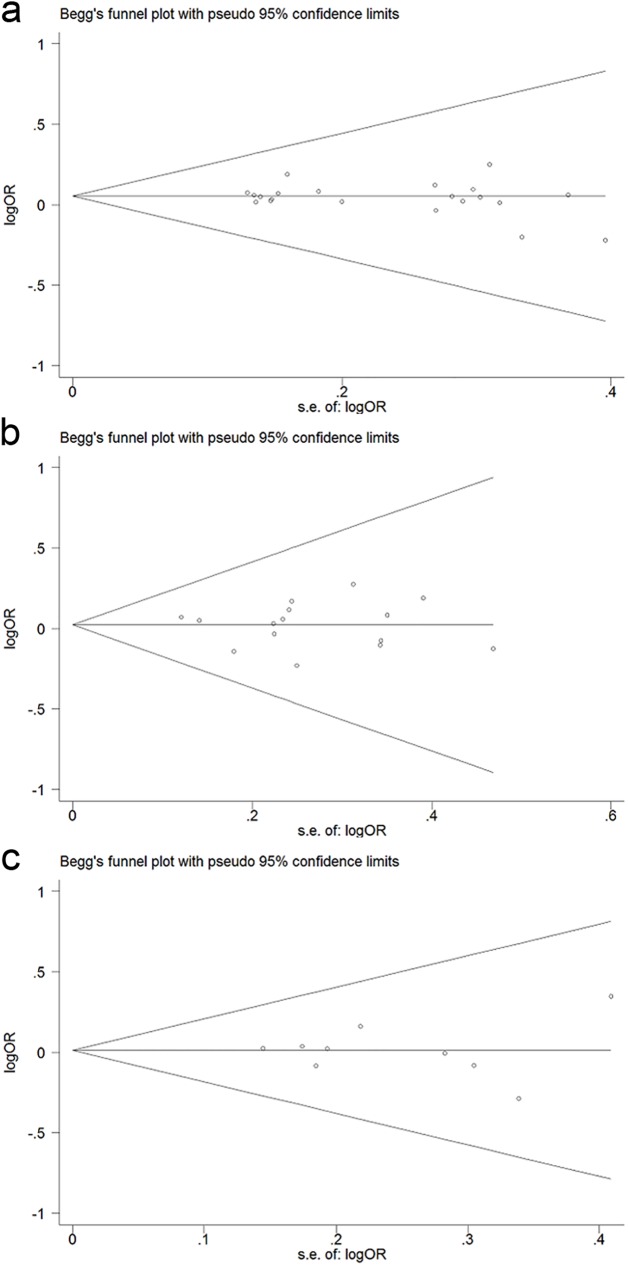
Begg’s funnel plot for publication bias under dominant model. (a) IL-1B-31C/T. (b) IL-1B-511-C/T. (c) IL-8-251T/A.

Begg’s funnel plot and Egger’s test were conducted to clarify potential publication bias. The shape of funnel plots showed a nearly symmetrical distribution, indicating no evidence of publication bias in all genetic models (Fig 6).Egger’s test also reveal no statistical significance for evaluation of publication bias under recessive model for *H*. *pylori* infection risk [IL-1B-31C/T: P = 0.275, IL-1B-511C/T: P = 0.06, IL-8-251A/T: P = 0.184], for H. pylori-related GC [IL-1B-511C/T: P = 0.781, IL-8-251A/T: P = 0.88], for *H*. *pylori*-related PUD [IL-8-251A/T: P = 0.71].

## Discussion

GC is one of the major causes of cancer-related death worldwide [61], and PUD is also a common disease worldwide with a lifetime prevalence in the adult population around 5% to 10% over the past decade [62]. It has been widely accepted that *H*. *pylori* infection is the most important etiological factor for the development of GC, and meanwhile plays a key roles in the pathogenesis of PUD. *H*. *pylori* infection is present in approximately 50% of the world population [63]. However, not every *H*. *pylori* infection individuals developed GC, PUD or other related diseases. In recent years, gene single nuclear polymorphisms (SNPs) have been identified as a very powerful tool for predicting some complex diseases. Prevalent inflammation related gene IL-1B and IL-8 polymorphisms may potentially alter the expression of them, which depress the secretion of gastric acid, amplify the inflammatory responses, thus being is favorable to *H*. *pylori* infection or *H*. *pylori*-related diseases. Increasing researchers have been focusing attention on the possible association between human inflammation related gene polymorphisms and *H*. *pylori* infection or *H*. *pylori*-related diseases. However, the associations have not been proved well. Therefore, we designed this meta-analysis to clarify the correlations between IL-1B31C/T, IL-1B-511C/T, IL-8-251T/A gene polymorphisms and the risk of *H*. *pylori* infection together with H. pylori-related GC and PUD.

Our results revealed that there is a significant difference in IL-1B-31C/T genotype distribution between *H*. *pylori* infection and control, and IL-1B-511C/T gene polymorphism might have no relation with the risk of *H*. *pylori* infection. For IL-1B-31C/T, the individuals’ susceptibility to H. pylori infection were increased in Asian population, but not in Caucasian population in the subgroup analysis, the frequency of IL-1B-31TT was higher among *H*. *pylori* infection than controls. Additionally, six studies with 647 *H*. *pylori*-related GC cases and 660 *H*. *pylori* inflection controls concerning IL-1B-511C/T gene polymorphisms, we found an increased risk of *H*. *pylori*-related GC with IL-1B-511C/T polymorphisms. And we found that different studies had inconsistent results about IL-1B-31C/T, IL-1B-511C/T gene polymorphism with the risk of *H*. *pylori* infection or *H*. *pylori*-related diseases. For instance, El-Omar EM et al[64] said that the polymorphisms of IL-1B-511T and IL-1B-31C alleles are significantly associated with *H*. *pylori* related diseases and the clinical presentations in a Caucasian research, they also concluded that IL-1B-31T allele is the wild type and the IL-1B-31C allele is a mutant, but Chang et al[65] said that mucosal IL-1β levels in *H*. *pylori*-infected GC patients were higher in patients homozygous for IL-1B-31T compared with IL-1B-31C/T and IL-1B-31C/C. To our knowledge, ethnic origin is a potent and key determinant of the frequency of genetic markers in a population which might well explain this mutation difference about IL-1B-31C/T gene in Asian and Caucasian population, which in turn means individuals with IL-1B-31TT genotype might have a high risk of *H*. *pylori* infection in Asian population. Another recent and new research investigated that *H*. *pylori* infection reinforces the relation between IL-1β-511 T allele with susceptibility to gastric cancer[OR = 2.04, 95%CI = 1.15–3.62 for recessive model], which means *H*. *pylori* infection as a risk factor might have synergistic effect with IL-1B-511T allele the development of GC[28].However, it exists a high Heterogeneity[P<0.0001, I^2^ = 84%]. In this meta-analysis, IL-1B-511TT genotype or T allele carriers might act as a potential candidate of biomarker for *H*. *pylori*-related GC risk [OR = 1.78, 95%CI = 1.29–2.47 for recessive model, p<0.001; OR = 1.77, 95%CI = 1.21–2.59 for TT vs CC model, p = 0.003], and there was no significant heterogeneity was observed between enrolled studies in all comparisons under each model[P>0.10,I^2^<50%]. Despite the decline in OR compared with the previous study, it is noteworthy that a significant risk between IL-1B-511C/T polymorphisms and *H*. *pylori*-related GC risk could be seen. But this result should be interpreted with caution because of limited studies and absence further research.

The meta-analysis of IL-8-251T/A included nine studies with 1963 cases and 1205 controls. No significant difference was found in IL-8-251T/A genotype distribution between *H*. *pylori* infection case and control, then we further conducted ethnic-based subgroup analysis, but there was no change in the association between IL-8-251T/A polymorphism and *H*. *pylori* infection. Interestingly, for IL-8-251T/A gene polymorphisms, three studies showed a significant increase risk of H. pylori-related GC, and five studies showed a significant increase risk *H*. *pylori*-related PUD. Although there was only limited studies in this meta-analysis, some new and recent researches came to support our results. For instance, Hofner et al [66] reported a higher frequency of the IL-8-251T/A genotype among *H*. *pylori*-related duodenal ulcer patients than controls. Another interesting Japanese research also indicated that the IL-8-251AA and AT genotypes might increase the individuals’ susceptibility to *H*. *pylori*-related diseases, but not increase the risk of *H*. *pylori* infection [9]. In terms of the mechanism, the allele A of IL-8-251T/A gene promoter region might increase the expression level of IL-8 gene, and the allele A carriers have a higher gastric mucosal neutrophil infiltration score, thus increasing the susceptibility to *H*. *pylori*-related diseases[67].Considering the above evidences, the association between IL-8-251T/A polymorphism and *H*. *pylori*-related diseases should be raised more attention, it’s necessary to conclude more case-control research to further clarify.

This research shed lights on the relationship of gene polymorphism and the increased susceptibility to H. pylori infection or H. pylori-related diseases systematically. The exhaustive inclusion criteria and studies combined with a series of subgroup analysis enhanced the power and persuasion of our conclusion. Additionally, all including literatures were consistent with HWE (P>0.05) and accepted as high quality (at least 6 score according to the NOS score). However, we were also aware of som e limitations of our study. Firstly, it is also possible that language bias might exist, as our meta-analysis only included articles published in Chinese, English and only one Korean. Secondly, the tendency not to publish negative results may also have produced bias. Thirdly, the number of studies, especially for the relationship of IL-8-251T/A polymorphism and H. pylori-related GC, was not sufficiently large, more studies involving much larger sampling sizes and well-designed character should be conducted.

## Conclusions

This meta-analysis indicated that IL-1B-31TT genotype might increase the individuals’ susceptibility to H. pylori infection, and this relationship was enhanced in Asian population. Additionally, IL-1B-511-C/T and IL-8-251T/A gene polymorphisms might act as a risk factor to H. pylori-related diseases including GC or PUD. Large and well-designed studies with different ethnicities are warranted to clarify our conclusions.

## Supporting information

S1 Checklist Meta-analysis on genetic association studies checklist. (DOCX)

S2 Checklist PRISMA checklist. (DOC)
